# Probiotic mixture VSL#3 reduce high fat diet induced vascular inflammation and atherosclerosis in ApoE^−/−^ mice

**DOI:** 10.1186/s13568-016-0229-5

**Published:** 2016-08-30

**Authors:** Yee Kwan Chan, Hani El-Nezami, Yan Chen, Kristiina Kinnunen, Pirkka V. Kirjavainen

**Affiliations:** 1 School of Biological Sciences, University of Hong Kong, Hong Kong, Hong Kong; 2 Institute of Public Health and Clinical Nutrition, University of Eastern Finland, Kuopio, Finland; 3 Department of Surgery, LKS Faculty of Medicine, University of Hong Kong, Hong Kong, Hong Kong; 4 Department of Health Protection, National Institute for Health and Welfare, PO Box 95, 70701 Kuopio, Finland

**Keywords:** Atherosclerosis, Gut microbiota, Vascular inflammation, VSL#3

## Abstract

Atherosclerosis results from chronic inflammation potentially caused by translocation of bacterial components from the oro-gastrointestinal tract to circulation. Specific probiotics have anti-inflammatory effects and may reduce bacterial translocation. We thereby tested whether a probiotic mixture with documented anti-inflammatory potential could reduce atherosclerosis. ApoE^−/−^ mice were fed high fat diet alone or with VSL#3 or a positive control treatment, telmisartan or both for 12 weeks. All treatments reduced atherosclerotic plaques significantly compared to high fat diet alone. VSL#3 significantly reduced proinflammatory adhesion molecules and risk factors of plaque rupture, reduced vascular inflammation and atherosclerosis to a comparable extent to telmisartan; and VSL#3 treated mice had the most distinctly different intestinal microbiota composition from the control groups. Combining the VSL#3 and telmisartan brought no further benefits. Our findings showed the therapeutic potential of VSL#3 in reducing atherosclerosis and vascular inflammation.

## Introduction

Atherosclerosis is recognized as an inflammatory disorder (Tedgui and Mallat 2006), its development of is a slow progress and can be contributed by non-modifiable parameters like gender, age, and genetics; as well as modifiable risk factors including sedentary lifestyle, smoking, etc. (Mercado and Jaimes 2007; Warren et al. 2010). Microbial immunogens originating from the gastrointestinal tract have been strongly associated as the key instigators of the inflammation that drives atherosclerotic development (Koren et al. 2010). Gut microbiota, the collection of 10^13^ to 10^14^ microbes that cover a thousand species, possess immunoregulatory functions, affects host’s energy harvest (Cani et al. 2008), lipid metabolism (Velagapudi et al. 2010) as well as the intestinal barrier integrity and thereby translocation of bacterial products (Sekirov et al. 2010). All these influences may indirectly affect the establishment of chronic inflammation. The essentiality of gut microbiota in the generation of atherogenic substances such as choline, trimethylamine N-oxide and betaine (Wang et al. 2011), shared bacterial phylotypes between atherosclerotic plaque and the oral and gut microbiota (Koren et al. 2011) and the specific gut microbial clustering and altered gut metagenome in atherosclerosis patients (Karlsson et al. 2012) all attest to the importance of gut bacteria in the pathogenesis of atherosclerosis.

The ability of specific probiotics to strengthen immunological and non-immunological gut barrier functions and thereby reduce translocation of bacterial and other immunogenic material from the gut has been reported (Mennigen and Bruewer 2009). In addition, there is some evidence associating the use of probiotics with reduction in several cardiovascular disease risk biomarkers, including serum total and LDL cholesterol (Guo et al. 2011), prevention of periodontal disease (Nase et al. 2001), and systemic inflammation (Kekkonen et al. 2008). VSL#3 is a well-studied probiotic mixture comprised of 8 strains of probiotics including *Bifidobacterium breve, Bifidobacterium longum, Bifidobacterium infantis, Lactobacillus acidophilus, Lactobacillus plantarum, Lactobacillus paracasei, Lactobacillus bulgaricus* and *Streptococcus thermophilus*. VSL#3 had been shown to confer beneficial effects to many disease conditions, including Crohn’s disease (Fedorak et al. 2015), ulcerative colitis (Mardini and Grigorian 2014) and liver disease (Dhiman et al. 2014). Recently, VSL#3 showed promising potential in the arena of atherosclerosis—it improved insulin signaling and protected against nonalcoholic steatohepatitis and atherosclerosis in ApoE^−/−^ mice with DSS-induced colitis (Mencarelli et al. 2012a); clinically, it significantly improved dyslipidemia profile in overweight adults (Lopez-Mejias et al. 2014) and in critically ill patients (Sanaie et al. 2013). Yet, the associations between the improvement in atherosclerosis and alteration in the gut microbes and gut hormones remain underexplored. Therefore, we examined the extent to how VSL#3 reduced atherosclerosis, the cardiovascular inflammation, and the effects of VSL#3 on the gut satiety hormones, inflammatory profile and the changes in the gut microbial community in apolipoprotein E knockout (ApoE^−/−^) mice, a well-established and popular model for atherosclerosis (Meir and Leitersdorf 2004). We compared the effects of VSL#3 with a positive control, telmisartan, an angiotensin II type I receptor blocker and partial agonists of perixosome proliferator-activated receptor-gamma (PPARg), which has been proved effective in reducing atherogenesis in this mouse model (Blessing et al. 2008). In addition, their combination effect was also assessed.

## Materials and methods

### Animals

Female 6-week-old apolipoprotein E knockout (ApoE^−/−^) mice (n = 4–5 per group) on C57B/6 background were fed a high-fat, western-type diet (D12079B, Research Diet Inc) alone (C), with telmisartan (1 mg/kg/day) (T1) or VSL#3 (2.78 × 10^11^ CFU/day) (V) or both (VT1) for 12 weeks. VSL#3 used was commercially available by VSL Pharmaceuticals Inc. Each unflavored VSL#3 sachet contained 4.5x10^11^ bacteria including *S. thermophilus*, *B. breve*, *B. bacterium longum*, *B. infantis*, *L. acidophilus*, *L. plantarum*, *L. paracasei* and *L. delbrueckii* subsp. *bulgarius*. The dosage of probiotics used in the study was based on the body surface area normalization method from the recommended human dose from VSL#3 (Reagan-Shaw et al. 2008). Telmisartan (Micardis^®^) (Boehringer Ingelheim GmbH) was administered to the mice in drinking water at 1 mg/kg/day (T1) with estimated mean water consumption at 15 ml/100 g/day. This dose has been previously shown to be effective in reducing atherosclerosis development in female ApoE^−/−^ mice (Takaya et al. 2006). All mice were kept in individually ventilated cages in Animal Laboratory of the Department of Surgery with regulated temperature from 23 to 24 °C and relative humidity at 60–70 % on 12/12 h day/night cycle. Weight and physical appearance was observed at least once a week. All the study protocols were approved by the Committee on the Use of Live Animals in Teaching and Research (CULATR) of the University of Hong Kong and the Department of Health of the HKSAR Government. Mice were fasted overnight before euthanization at the end of week 12.

### Atherosclerosis lesion quantification

At the end of the experiment, heart was perfused with PBS followed by 4 % paraformaldehyde at the time of sacrifice to remove blood and for initial fixation. It was further fixed in 4 % paraformaldehyde overnight before embedding in Tissue Tek^®^ O.C.T compound (Sakura, Finetek USA Inc.) and stored at −20 °C until use. The heart was cryosectioned serially at 10 µm intervals from the aortic sinus and mounted on slides (Superfrost plus, Thermo Fisher Scientific Inc.). Oil Red O solution was freshly prepared as described in Baglione and Smith (Baglione and Smith 2006) and used within 2 h of preparation. Bluing solution was prepared by adding five drops of ammonia in 1000 ml ddH_2_O. The slides were immersed in ddH_2_O for 2 min; 60 % isopropanol for 30 s; Oil Red O solution for 18 min; 60 % isopropanol for 30 s; ddH_2_O for 1 min; ddH_2_O for 1 min; hematoxylin (Vector^®^ Laboratory Inc., CA 94010 USA) for 2 min; Bluing solution for 10 s; ddH_2_O for 1 min; Methyl Green (Dako, DK-2600 Glostrup Demark) for 1 min and ddH_2_O for 15 min before air drying. The sections were covered with Faramount aqueous mounting medium (Dako, DK-2600 Glostrup Demark). Intimal lesion area was quantified by averaging at least 10 sections spaced 30 µm apart from the base of the aortic root. Images were viewed and captured with Nikon ultra-high-quality digital camera DXM1200F and acquired with Nikon ACT-1 software (version 2.60). Lesion area was analyzed using Image J software.

### Plasma atherosclerotic biomarkers quantification

At the end of experiment the mice were fasted overnight and blood was taken from posterior vene cava and stored at −80 °C until use with less than 2 freeze-thaw cycles. The concentrations of pancreatic polypeptide (PP), peptide YY (PYY), glucagon-like peptide 1 (GLP-1), monocyte chemoattractant protein-1 (MCP-1), tumor necrosis factor (TNF), soluble E-selectin (sE-selectin), soluble vascular cell adhesion molecule-1 (sVCAM-1), soluble inter-cellular adhesion molecule-1 (sICAM-1), fibrinogen, matrix metalloproteinase-9 (MMP-9) and total plasminogen activator inhibitor-1 (tPAI-1) were quantified using MILLIPLEX Mouse multiplex kits (Millipore Corporation, USA) and Bio-Plex™ 200 System (Bio-Rad Laboratories, Hercules, CA), following the manufacturer’s protocol in a 96-well plate format.

### Gut microbiota profiling. DNA isolation

The ileum and colon were collected at the end of experiment, freeze dried with liquid nitrogen before storing at −80 °C until use. Genomic DNA of the intestinal wall scrapings and luminal content was extracted using NucleoSpin^®^ Tissue (Macherey–Nagel GmbH & Co. KG, Dϋren, Germany) according to the user manual and stored at −20 °C before use.

### Primers and PCR conditions

Primers for universal bacterial (U968-GC: GC clamp–AACGCGAAGAACCTTAC; L141-Cy5: Cy5-GCGTGTGTACAAGACCC) (Nubel et al. 1996; Randazzo et al. 2006) were used to amplify the V6 to V8 regions of the bacterial 16S rRNA of the respective bacteria in ileum and colon. Primers were synthesized by Oligomer Oy (Helsinki, Finland). The GC clamp attached at the 5′ end of one of the primers made the products more suitable for separation by temperature gradient gel electrophoresis (TGGE) afterwards. PCR was performed using the GoTaq^®^ Flexi DNA polymerase kit (Promega Corporation, USA). The PCR condition for Universal bacteria was: PCR mixtures of 50 µl containing 1× Go Tag Buffer, 3 mM MgCl_2_, 0.05 mM dNTP, 5 µM of respective forward and reverse primers, 1.25 U of Go Tag Polymerase and 20–50 ng of the isolated template genomic DNA. Samples were amplified with Biometra T3 Thermocycler (Golndustry DoveBid, D-37079 Goettingen, Germany) by using the following program: predenaturation at 94 °C for 5 min; 35 cycles of denaturation at 94 °C for 30 s, annealing temperature at 56 °C for 30 s and extension at 72 °C for 40 s; and a final extension at 72 °C for 7 min.

### TGGE analysis of PCR amplicons

PCR products generated with above mentioned primers were separated by TGGE by using Biometra TGGE MAXI system (Biomedizinische Analytik GmbH, Germany). PCR samples were applied directly onto 8 % (wt/vol) acrylamid stock solutions (acrylamide-N,N’-methylenebisacrylamide 37.5:1) gels with 8 M urea, 20 % formamide, 2 % glycerol and 1× TBE where polymeraization was started by adding 80 µl 10 % APS and 100 µl 100 % TEMED quickly. Electrophoreses was performed at 20 °C at 300 V for 15 min; 42–52 °C at 0 V for 10 min; 42–52 °C at 150 V for 18 h and 42–52 °C at 25 V for 4 h in 0.5× TBE running buffer. The gel was read using red excited fluorescence in Typhoon™ (GE Healthcare FIN-00002 Helsinki, Finland) with Storm™ ImageQuant analysis software (GE Healthcare FIN-00002 Helsinki, Finland).

### Statistical analysis

Generally graphs are presented as mean ± SEM unless otherwise stated. Significance were tested by one way analysis with GraphPad Prism software, with significance assumed at p < 0.05.

## Results

### High fat diet induced atherosclerotic lesion was reduced by VSL#3, telminsartan and its combination

**Fig. 1:**
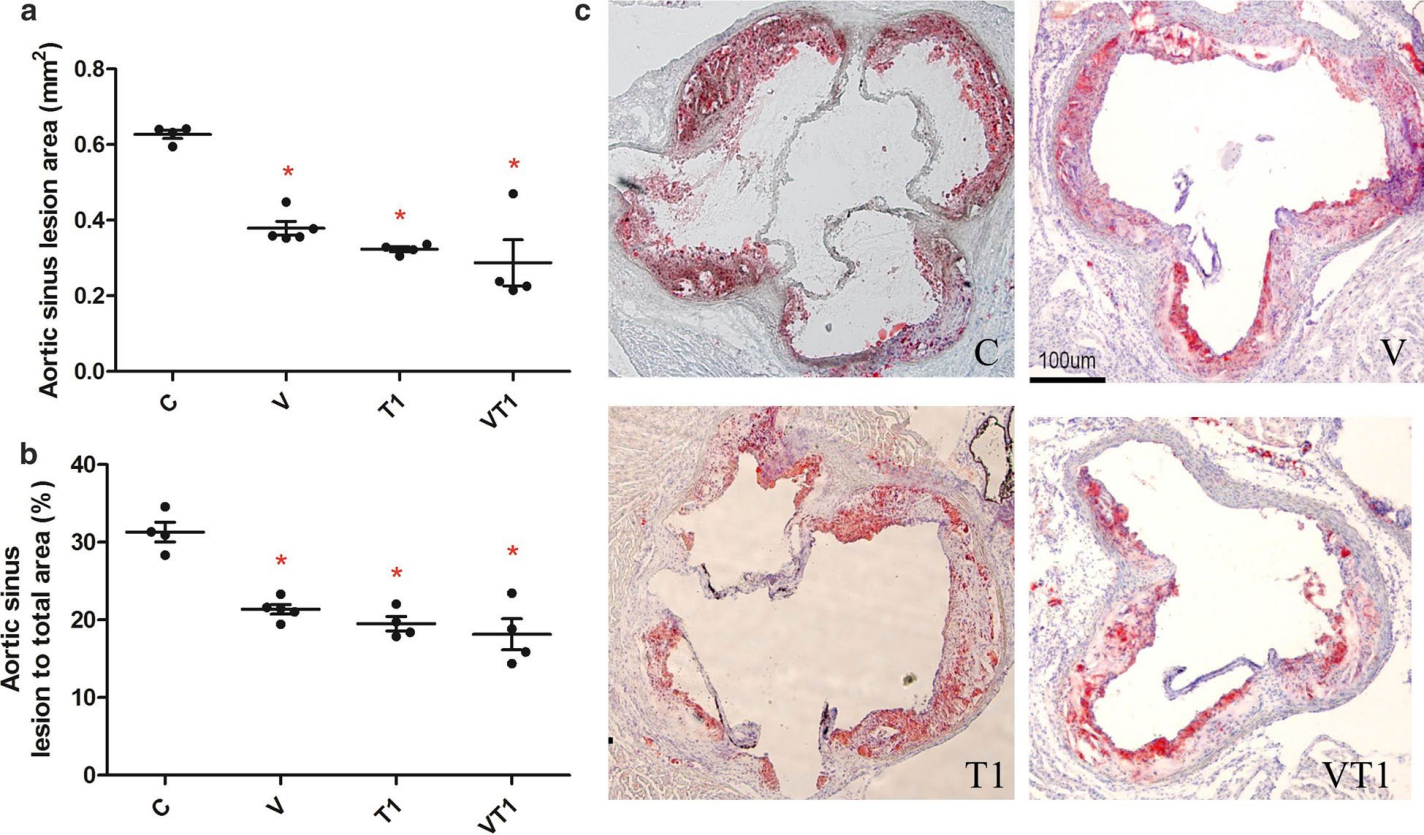
Atherosclerotic lesion characterization. ApoE^−/−^ mice (n = 4–5 per group) were fed high fat diet (*C*) supplemented with VSL#3 (*V*), telmisartan (*T1*) or both (*VT1*). Quantification of lesion at aortic sinus in absolute area (**a**), percentage area (**b**) and lipid staining of lesion with Oil Red O (**c**). Data was presented as mean ± SD. *Asterisk* indicates significant differences from the control group (**c**). *p < 0.05

High fat diet fed mice had significant fat deposition in the aortic root at 0.63 ± 0.02 mm^2^, which was significantly reduced by the supplementation of VSL#3 (0.38 ± 0.04 mm^2^), T1 (0.32 ± 0.01 mm^2^) and its combination (0.29 ± 0.12 mm^2^) (Fig. 1a). Similar results were shown in terms of the percentage of atherosclerotic lesion over the total aortic sinus area (Fig. 1b). While VSL#3 reduced the lesion area at similar extent to the positive control telminsartan, their combination did not lead to further lesion area reduction.

### VSL#3 significantly increased satiety hormone PYY without altering weight change and food intake

**Fig. 2:**
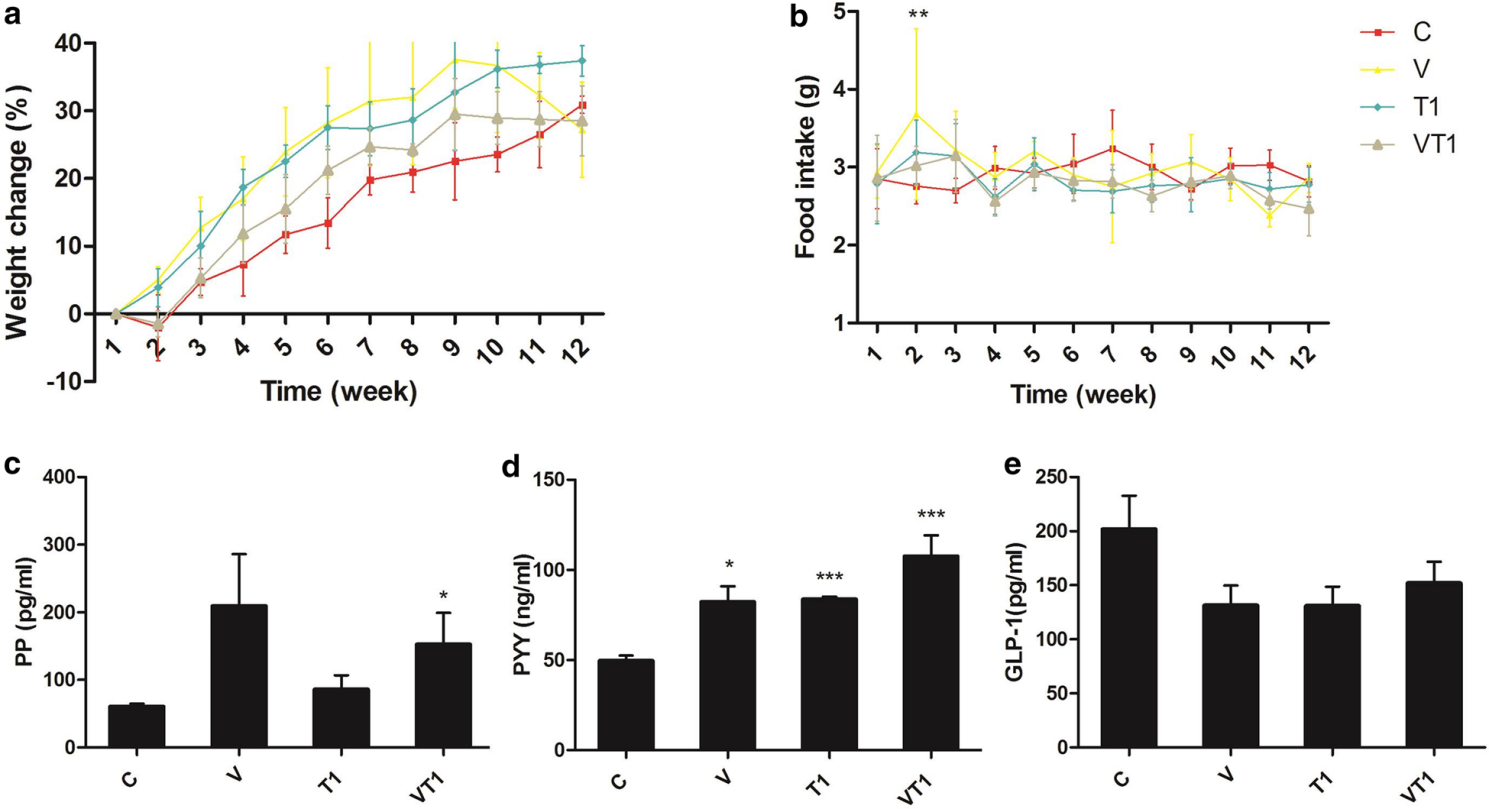
Mice weight change, food intake and gut hormones levels at week 12. ApoE^−/−^ mice (n = 4–5 per group) were fed high fat diet (*C*) supplemented with VSL#3 (*V*), telmisartan (*T1*) or both (*VT1*). Weight change (**a**), food intake (**b**), serum PP (**c**), PYY (**d**) and GLP-1 (**e**). Data was presented as mean ± SD. *Asterisk* indicates significant differences from the control group (*C*). *p < 0.05; **p < 0.01; ***p < 0.001

The influence of VSL#3 to satiety hormones were studied by quantifying the two most well- known members, pancreatic polypeptide (PP) and polypeptide YY (PYY). VSL# tended to increase the satiety hormone pancreatic polypeptide (PP), but such increase was only significant with the combination of T1 (Fig. 2a). On the other hand, all treatment groups had resulted in a significant increase in PYY (Fig. 2b). Yet, weight change and food intake was similar among all groups (Fig. 2c, d) It suggests that the changes observed in atherosclerotic lesions, biomarkers or gut microbiota were not associated with changes of food intake or weight gain as neither of the parameters were consistently affected by the treatments.

### VSL#3 reduced vascular inflammation

**Fig. 3:**
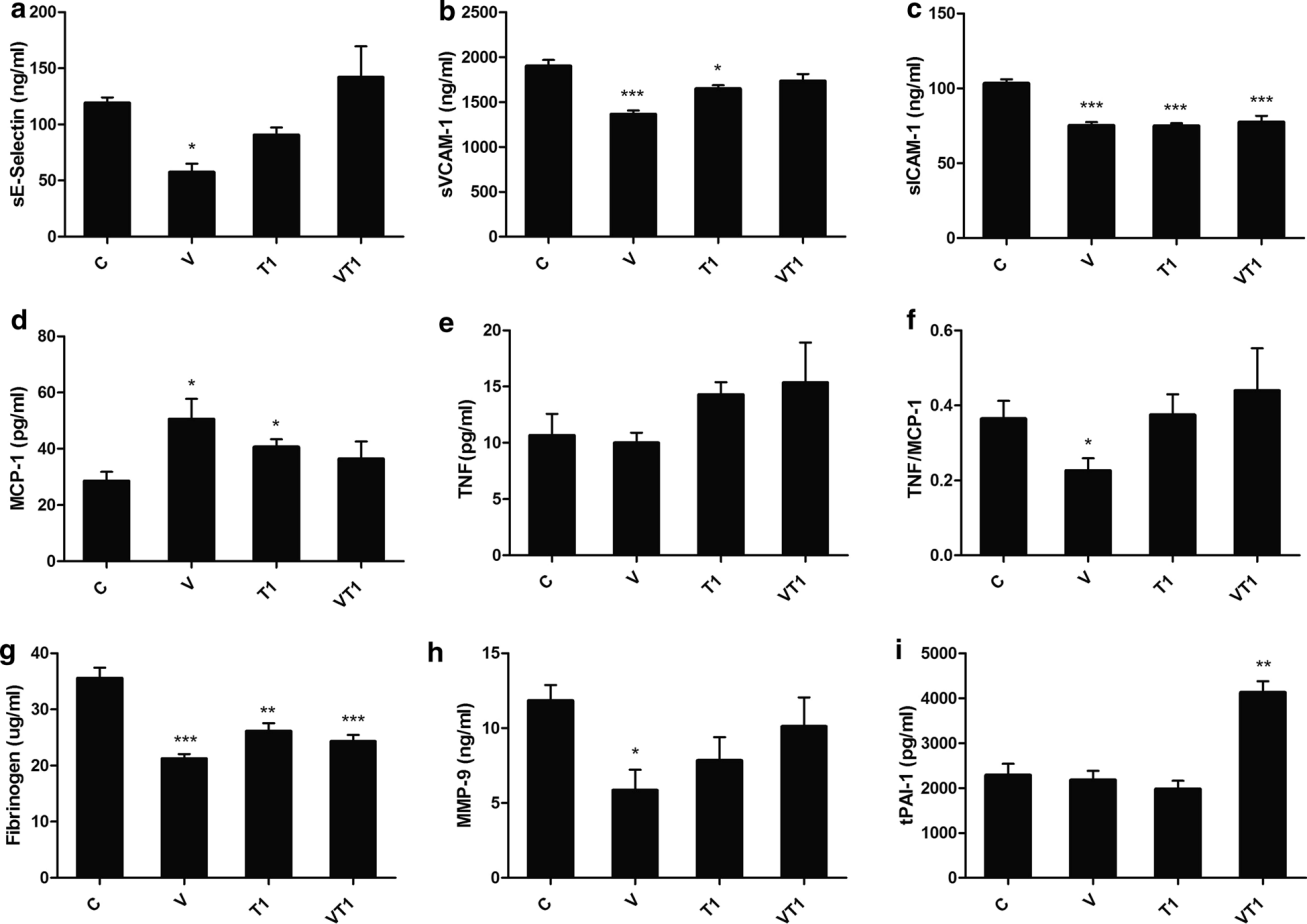
Atherosclerosis related biomarkers in plasma. ApoE^−/−^ mice (n = 4–5 per group) were fed high fat diet (*C*) supplemented with VSL#3 (*V*), telmisartan (*T1*) or both (*VT1*). Serum sE-selectin (**a**), sVCAM-1 (**b**), sICAM-1 (**c**), MCP-1 (**d**), TNF (**e**), TNF/MCP-1 (**f**), fibrinogen (**g**), MMP-9 (**h**) and tPAI-1 (**i**). Data was presented as mean ± SD. *Asterisk* indicates significant differences from the control group (**c**). *p < 0.05; **p < 0.01; ***p < 0.001

All supplementations were effective in lowering adhesion molecules but in different extents—VT1 lowered sICAM-1 significantly, T1 lowered sICAM-1 and sVCAM-1, while V lowered sICAM-1, sVCAM-1 and sE-selectin (Fig. 3a–c). While V and T1 increased the macrophage recruiting MCP-1 levels, the TNF levels were not significantly altered, resulting in an overall decrease of TNF to MCP-1 ratio by V (Fig. 3d). All treatment groups resulted in significantly lower fibrinogen; while V significantly lowered MMP-9, T1 and VT1 only showed similar tendency. VT1, on the other hand significantly elevated tPAI-1 (Fig. 3g–i).

### VSL#3 resulted in distinct ileal and colonic microbial profile with increased diversity

**Fig. 4:**
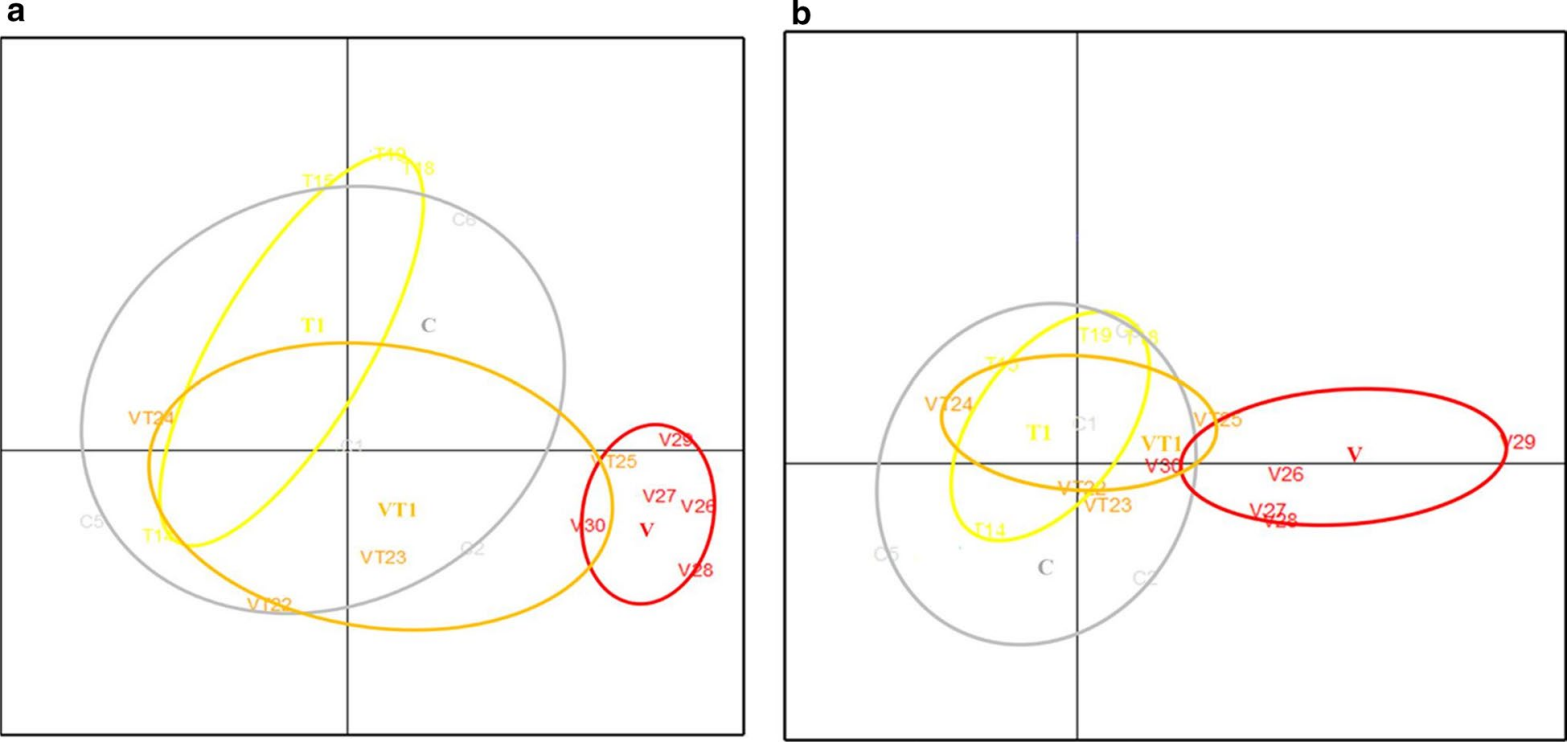
Colonic microbial profile. ApoE^−/−^ mice were fed high fat diet (*C*) supplemented with VSL#3(*V*), telmisartan (*T1*) or both (*VT1*). Overall similarity of dominant bacterial composition in the ileum (**a**) and colon (**b**) of different mice (*numbers*) and clustering by treatment (*ellipses*) as visualized by PCA analysis. Clustering indicates *high* and separation *low* compositional similarity between the different mice

All treatments affected the dominant bacterial composition in the GIT from the changes in the TGGE-banding profiles. Furthermore, VSL#3 had led to the appearance of three distinct bacterial banding that was absent in the control group. When these bandings were mapped into a principle coordinate analysis plot, it became obvious that different treatments rendered a new clustering of microbes (Fig. 4a, b). Supplementation with VSL#3 alone resulted in distinct compositional profile from the control while VT1 showed a partial change towards VSL#3-like profile. Different supplementations resulted in similar microbial changes in the ileum and colon (Fig. 4).

## Discussion

VSL#3 reduced the high fat diet induced lesion development in aortic root in comparable manner to the positive control drug, telmisartan (Fig. 1). VSL#3 is recently proved protective against the development of steotohepatitis and atherosclerosis in the same model with dextran sulfate sodium-induced intestinal inflammation (Mencarelli et al. 2012b), yet, another study with the same model, supplementation of high fat diet with other probiotics (*L. reuteri* strains, ATCC 4659, DSM or L6798) did not result lesion area reduction at aortic sinus (Fak and Backhed 2012). Our study has further proved the efficacy of VSL#3 in reducing atherosclerotic plaque development, drawing importance to the alteration of gut microbial profile.

Comparable reduction in lesion develoment as in the current study, has been previously achieved with prebiotic inulin supplementation in ApoE^−/−^ mice (Rault-Nania et al. 2006). Interestingly, inulin and VSL#3 are both bifidogenic, i.e. reinforce the endogenous bifidobacterial microbiota. In addition, VSL#3 contains three *Bifidobacterium* species itself (Kuhbacher et al. 2006). Thus, it is possible that bifidobacteria or at least some specific *Bifidobacterium* species may be particularly proficient in host–gut microbe interaction mediated prevention of atherosclerosis (Kolida et al. 2002). The idea is further supported by a historical cohort study, suggesting that breast-feeding, which is known for its robust bifidogenic effect, may offer protection against atherosclerosis development later in life (Martin et al. 2005). These findings are also in line with the bacterial translocation theory of atherosclerosis, considering that both, bifidobacteria and breast-feeding, are well-known for their gut barrier fortifying effects and reduction of bacterial translocation (Lievin et al. 2000).

With the known anti-inflammatory property of VSL#3, we proceeded to investigate if VSL#3 could reduce inflammation in the high fat diet fed ApoE^−/−^ mice. Adhesion molecules were lowered by all supplementations but at different extents. A significant lowered level of adhesion molecules may represent a lower risk of the development of endothelial dysfunction and formation of atherosclerotic plaque. In particular, the aortic concentration of VCAM and ICAM had been confirmed to be lowered by VSL#3 (Mencarelli et al. 2012a). It is interesting that VSL#3 reduced all adhesion molecules and biomarkers of inflammation, as it reflected that the plaque vulnerability maybe reduced. Furthermore, the gelatinase MMP-9 lowered by VSL#3 indicated its potential association with lowered risk of thrombotic events, for example stroke and ischemia. Surprisingly, another atherosclerosis development and thrombosis associated biomarker, tPAI-1, was significantly increased by telmisartan with VSL#3 as combined supplement but not by either of the treatments alone. This was unexpected as, while angiotensin II can lead to increase synthesis of PAI-1 (Munger 2011), as its blocker, telmisartan should theoretically be capable in lowering PAI-1 or prevent its increase (Hasegawa et al. 2011). Similarly, although the effects of VSL#3 are unknown, other probiotics and culture strains have been shown to produce bioactive peptides that may increase the inhibitory functions of angiotensin-converting enzyme and thus formation of angiotensin II (Lye et al. 2009). Why the combination of the two treatments then led to increase in tPAI-1 remains obscure. Telmisartan also appeared to interfere with some of the VSL#3-mediated anti-inflammatory effects seen as significantly lower serum sVCAM-1, sE-selectin and MMP-9 concentrations with VSL#3 alone than with V + T1. The mechanism of such interference will require further studies. Both VSL#3 and telmisartan have been previously linked with PPARg mediated mechanisms (Matsumura et al. 2011). However, whereas VSL#3 has been associated with reduced gut epithelial permeability and thus reduced antigen translocation induced inflammation, telmisartan may increase endothelial permeability (Bian et al. 2009). Also, as discussed below, the microbiota development was affected differently by probiotics alone than probiotics in conjunction with telmisartan. Nevertheless, despite overriding many of the effects seen with VSL#3 alone, the combined treatment seemed at least as effective, if not better, in reducing the plaque development than the two treatments alone. This could indicate irrelevance of the observed changes in the proinflammatory biomarkers and gut microbiota, regarding the lesion development hindrance for both of the treatments or merely that of telmisartan.

After confirming the anti-inflammatory impact of VSL#3 in high fat diet fed ApoE^−/−^ mice, we further studied if VSL#3 alter the colonic microbial profiles. The universal bacteria from the colon were amplified and separated on an acrylamide gel based on their sizes. The TGGE banding patterns of the preliminary study of the gut microbiota indicated that VSL#3 supplementation had the most characterizable influence on the predominant microbiota composition in the colon. Notably, in combination with telmisartan the effect of VSL#3 seemed more moderate although the within-group similarity remained higher than seen within the control and other treatment groups (Fig. 4). The mechanism behind the effects of telmisartan on microbiota can only be speculated upon but could reflect the selective antimicrobial potential previously associated with telmisartan (Kruszewska et al. 2002). While the causal relationship to atherosclerosis inhibition is not known, these crude profiles of the predominant endogenous gut bacteria indicate that further studies with more thorough methodology are warranted to investigate whether the beneficial effects of VSL#3 were mediated via compositional modulation of gut microbiota. The appearance of various bacterial bands in association with VSL#3, as contrast to high fat diet alone, suggests VSL#3 may have increased diversity in the gut. In this respect it is notable that an intake of probiotic *L. plantarum* was shown to diversify the gut microbiota in men with incipient atherosclerosis (Karlsson et al. 2010). While the effects on atherosclerosis per se were not assessed in that study, the diversification could be beneficial since disorders with pro-inflammatory component tend to be associated with microbial composition with reduced diversity, as in inflammatory bowel disease where VSL#3 has been associated with beneficial effects and also ability of diversify the microbiota.

In conclusion, VSL#3 reduced biomarkers of vascular inflammation and development of atherosclerosis to comparable extent as the positive control drug, telmisartan in ApoE^−/−^ mice. Further studies in human subjects are warranted to verify the findings.

## Abbreviations

ApoE: apolipoprotein E
DSS: dextran sulphate sodium
GLP-1: glucagon-like peptide-1
LDL: low density lipoprotein
MCP-1: monocyte chemoattractant protein-1
MMP-9: matrix metalloproteinase-9
PP: pancreatic polypeptide
PPARg: peroxisome proliferator activated receptor-gamma
PYY: peptide YY
sE-selectin: soluble E-selectin
sICAM-1: soluble inter-cellular adhesion molecule-1
sVCAM-1: soluble vascular cell adhesion molecule-1
TGGE: temperature gradient gel electrophoresis
TNF: tumor necrosis factor
tPAI-1: total plasminogen activator inhibitor-1

## References

[CR1] Baglione J, Smith JD (2006). Quantitative assay for mouse atherosclerosis in the aortic root. Methods Mol Med.

[CR2] Bian C, Wu Y, Chen P (2009). Telmisartan increases the permeability of endothelial cells through zonula occludens-1. Biol Pharm Bull.

[CR3] Blessing E, Preusch M, Kranzhofer R, Kinscherf R, Marx N, Rosenfeld ME, Isermann B, Weber CM, Kreuzer J, Grafe J, Katus HA, Bea F (2008). Anti-atherosclerotic properties of telmisartan in advanced atherosclerotic lesions in apolipoprotein E deficient mice. Atherosclerosis.

[CR4] Cani PD, Delzenne NM, Amar J, Burcelin R (2008). Role of gut microflora in the development of obesity and insulin resistance following high-fat diet feeding. Pathol Biol.

[CR5] Dhiman RK, Rana B, Agrawal S, Garg A, Chopra M, Thumburu KK, Khattri A, Malhotra S, Duseja A, Chawla YK (2014). Probiotic VSL#3 reduces liver disease severity and hospitalization in patients with cirrhosis: a randomized, controlled trial. Gastroenterology.

[CR6] Fak F, Backhed F (2012). Lactobacillus reuteri prevents diet-induced obesity, but not atherosclerosis, in a strain dependent fashion in Apoe-/- mice. PLoS ONE.

[CR7] Fedorak RN, Feagan BG, Hotte N, Leddin D, Dieleman LA, Petrunia DM, Enns R, Bitton A, Chiba N, Pare P, Rostom A, Marshall J, Depew W, Bernstein CN, Panaccione R, Aumais G, Steinhart AH, Cockeram A, Bailey RJ, Gionchetti P, Wong C, Madsen K (2015). The probiotic VSL#3 has anti-inflammatory effects and could reduce endoscopic recurrence after surgery for Crohn’s disease clinical gastroenterology and hepatology: the official clinical practice. J Am Gastroenterol Assoc.

[CR8] Guo Z, Liu XM, Zhang QX, Shen Z, Tian FW, Zhang H, Sun ZH, Zhang HP, Chen W (2011). Influence of consumption of probiotics on the plasma lipid profile: a meta-analysis of randomised controlled trials. Nutr Metab Cardiovasc Dis.

[CR9] Hasegawa H, Takano H, Narumi H, Ohtsuka M, Mizuguchi T, Namiki T, Kobayashi Y, Komuro I (2011). Effects of telmisartan and losartan on cardiovascular protection in Japanese hypertensive patients hypertension research: official journal of the Japanese Society of. Hypertension.

[CR10] Karlsson C, Ahrne S, Molin G, Berggren A, Palmquist I, Fredrikson GN, Jeppsson B (2010). Probiotic therapy to men with incipient arteriosclerosis initiates increased bacterial diversity in colon: a randomized controlled trial. Atherosclerosis.

[CR11] Karlsson FH, Fak F, Nookaew I, Tremaroli V, Fagerberg B, Petranovic D, Backhed F, Nielsen J (2012). Symptomatic atherosclerosis is associated with an altered gut metagenome. Nat Commun.

[CR12] Kekkonen RA, Lummela N, Karjalainen H, Latvala S, Tynkkynen S, Jarvenpaa S, Kautiainen H, Julkunen I, Vapaatalo H, Korpela R (2008). Probiotic intervention has strain-specific anti-inflammatory effects in healthy adults. World J Gastroenterol.

[CR13] Kolida S, Tuohy K, Gibson GR (2002). Prebiotic effects of inulin and oligofructose. Br J Nutr.

[CR14] Koren O, Spor A, Felin J, Fak F, Stombaugh J, Tremaroli V, Behre CJ, Knight R, Fagerberg B, Ley RE, Backhed F (2010). Human oral, gut, and plaque microbiota in patients with atherosclerosis. Proc Natl Acad Sci USA.

[CR15] Koren O, Spor A, Felin J, Fak F, Stombaugh J, Tremaroli V, Behre CJ, Knight R, Fagerberg B, Ley RE, Backhed F (2011). Human oral, gut, and plaque microbiota in patients with atherosclerosis. Proc Natl Acad Sci USA.

[CR16] Kruszewska H, Zareba T, Tyski S (2002). Search of antimicrobial activity of selected non-antibiotic drugs. Acta Pol Pharm.

[CR17] Kuhbacher T, Ott SJ, Helwig U, Mimura T, Rizzello F, Kleessen B, Gionchetti P, Blaut M, Campieri M, Folsch UR, Kamm MA, Schreiber S (2006). Bacterial and fungal microbiota in relation to probiotic therapy (VSL#3) in pouchitis. Gut.

[CR18] Lievin V, Peiffer I, Hudault S, Rochat F, Brassart D, Neeser JR, Servin AL (2000). Bifidobacterium strains from resident infant human gastrointestinal microflora exert antimicrobial activity. Gut.

[CR19] Lopez-Mejias R, Genre F, Garcia-Bermudez M, Ubilla B, Castaneda S, Llorca J, Gonzalez-Juanatey C, Corrales A, Miranda-Filloy JA, Pina T, Gomez-Vaquero C, Rodriguez-Rodriguez L, Fernandez-Gutierrez B, Balsa A, Pascual-Salcedo D, Lopez-Longo FJ, Carreira P, Blanco R, Martin J, Gonzalez-Gay MA (2014). Lack of association between ABO, PPAP2B, ADAMST7, PIK3CG, and EDNRA and carotid intima-media thickness, carotid plaques, and cardiovascular disease in patients with rheumatoid arthritis. Mediators Inflamm.

[CR20] Lye HS, Kuan CY, Ewe JA, Fung WY, Liong MT (2009). The improvement of hypertension by probiotics: effects on cholesterol, diabetes, renin, and phytoestrogens. Int J Mol Sci.

[CR21] Mardini HE, Grigorian AY (2014). Probiotic mix VSL#3 is effective adjunctive therapy for mild to moderately active ulcerative colitis: a meta-analysis. Inflamm Bowel Dis.

[CR22] Martin RM, Ebrahim S, Griffin M, Davey Smith G, Nicolaides AN, Georgiou N, Watson S, Frankel S, Holly JM, Gunnell D (2005). Breastfeeding and atherosclerosis: intima-media thickness and plaques at 65-year follow-up of the Boyd Orr cohort. Arterioscler Thromb Vasc Biol.

[CR23] Matsumura T, Kinoshita H, Ishii N, Fukuda K, Motoshima H, Senokuchi T, Taketa K, Kawasaki S, Nishimaki-Mogami T, Kawada T, Nishikawa T, Araki E (2011). Telmisartan exerts antiatherosclerotic effects by activating peroxisome proliferator-activated receptor-gamma in macrophages. Arterioscler Thromb Vasc Biol.

[CR24] Meir KS, Leitersdorf E (2004). Atherosclerosis in the apolipoprotein-E-deficient mouse: a decade of progress. Arterioscler Thromb Vasc Biol.

[CR25] Mencarelli A, Cipriani S, Renga B, Bruno A, D’Amore C, Distrutti E, Fiorucci S (2012). VSL#3 resets insulin signaling and protects against NASH and atherosclerosis in a model of genetic dyslipidemia and intestinal inflammation. PLoS ONE.

[CR26] Mencarelli A, Cipriani S, Renga B, Bruno A, D’Amore C, Distrutti E, Fiorucci S (2012). VSL#3 resets insulin signaling and protects against NASH and atherosclerosis in a model of genetic dyslipidemia and intestinal inflammation. PLoS ONE.

[CR27] Mennigen R, Bruewer M (2009). Effect of probiotics on intestinal barrier function. Ann N Y Acad Sci.

[CR28] Mercado C, Jaimes EA (2007). Cigarette smoking as a risk factor for atherosclerosis and renal disease: novel pathogenic insights. Curr Hypertens Rep.

[CR29] Munger MA (2011). Use of angiotensin receptor blockers in cardiovascular protection: current evidence and future directions. PT.

[CR30] Nase L, Hatakka K, Savilahti E, Saxelin M, Ponka A, Poussa T, Korpela R, Meurman JH (2001). Effect of long-term consumption of a probiotic bacterium *Lactobacillus rhamnosus* GG, in milk on dental caries and caries risk in children. Caries Res.

[CR31] Nubel U, Engelen B, Felske A, Snaidr J, Wieshuber A, Amann RI, Ludwig W, Backhaus H (1996). Sequence heterogeneities of genes encoding 16S rRNAs in *Paenibacillus polymyxa* detected by temperature gradient gel electrophoresis. J Bacteriol.

[CR32] Randazzo CL, Vaughan EE, Caggia C (2006). Artisanal and experimental Pecorino Siciliano cheese: microbial dynamics during manufacture assessed by culturing and PCR-DGGE analyses. Int J Food Microbiol.

[CR33] Rault-Nania MH, Gueux E, Demougeot C, Demigne C, Rock E, Mazur A (2006). Inulin attenuates atherosclerosis in apolipoprotein E-deficient mice. Br J Nutr.

[CR34] Reagan-Shaw S, Nihai M, Ahmad N (2008). Dose translation from animal to human studies revisited. FASEB J.

[CR35] Sanaie S, Ebrahimi-Mameghani M, Mahmoodpoor A, Shadvar K, Golzari SE (2013). Effect of a probiotic preparation (VSL#3) on cardiovascular risk parameters in critically-ill patients. J Cardiovasc Thorac Res.

[CR36] Sekirov I, Russell SL, Antunes LC, Finlay BB (2010). Gut microbiota in health and disease. Physiol Rev.

[CR37] Takaya T, Kawashima S, Shinohara M, Yamashita T, Toh R, Sasaki N, Inoue N, Hirata K, Yokoyama M (2006). Angiotensin II type 1 receptor blocker telmisartan suppresses superoxide production and reduces atherosclerotic lesion formation in apolipoprotein E-deficient mice. Atherosclerosis.

[CR38] Tedgui A, Mallat Z (2006). Cytokines in atherosclerosis: pathogenic and regulatory pathways. Physiol Rev.

[CR39] Velagapudi VR, Hezaveh R, Reigstad CS, Gopalacharyulu P, Yetukuri L, Islam S, Felin J, Perkins R, Boren J, Oresic M, Backhed F (2010). The gut microbiota modulates host energy and lipid metabolism in mice. J Lipid Res.

[CR40] Wang Z, Klipfell E, Bennett BJ, Koeth R, Levison BS, Dugar B, Feldstein AE, Britt EB, Fu X, Chung YM, Wu Y, Schauer P, Smith JD, Allayee H, Tang WH, DiDonato JA, Lusis AJ, Hazen SL (2011). Gut flora metabolism of phosphatidylcholine promotes cardiovascular disease. Nature.

[CR41] Warren TY, Barry V, Hooker SP, Sui X, Church TS, Blair SN (2010). Sedentary behaviors increase risk of cardiovascular disease mortality in men. Med Sci Sports Exerc.

